# From Living Systematic Reviews to Fully AI-Driven Reviews

**DOI:** 10.7759/cureus.113428

**Published:** 2026-07-26

**Authors:** Zubair Mojadeddi, Jason J Baker, Jacob Rosenberg

**Affiliations:** 1 Department of Surgery, Center for Perioperative Optimization, Herlev and Gentofte Hospital, University of Copenhagen, Herlev, DNK

**Keywords:** artificial intelligence in medicine, living systematic reviews, medical education, research methodology and ethics, systematic reviews and meta-analyses

## Abstract

Artificial intelligence (AI) holds great promise for improving systematic reviews, particularly as the number of reviews keeps growing. A solution to keep systematic reviews up to date are the so-called living systematic reviews. Although they could be a solution, they currently face challenges regarding everything having to be done manually. A potential solution could be AI-driven reviews that automate each step, from literature searching and translation to data extraction and analysis. Potentially, AI could search through large amounts of studies, sort them by relevance, and extract relevant data. This significantly reduces the workload for researchers, and thereby gives researchers time to focus on oversight rather than manual tasks. However, these methods raise concerns about algorithmic bias and transparency. Proper training, clear ethical guidelines, and interdisciplinary collaboration are keys to ensuring quality and integrity. AI-driven reviews may become essential for efficiently handling the expanding scientific literature.

## Editorial

Introduction

Artificial intelligence (AI) has received increasing attention over the past few years, such as in the writing processes, brainstorming, and searching for patient data in medical records, among other tasks. One promising area for AI integration is optimizing systematic reviews. Over the past decade, the number of systematic reviews has grown, and a recent study found that publications of systematic reviews increased continuously with a median annual growth rate of 26%, while publication of Cochrane reviews decreased since 2015 [1]. This growth highlights the need for more efficient methods to produce, update, and disseminate reviews. Furthermore, the increase in original studies has influenced the growth of systematic reviews and intensified the challenges of keeping them up to date in rapidly evolving fields. In response, "living systematic reviews” were introduced as a new approach to ensure that systematic reviews remain current and continuously incorporate new evidence [2]. The concept of "living systematic reviews” builds on the idea that systematic reviews, in collaboration with technology, could be automated and continually updated with relevant and new knowledge [2], thereby optimizing and streamlining the production of reviews without compromising methodology. However, despite the existence of living systematic reviews, they do not fully address the idea of being continuously updated because the processes are currently conducted manually. Therefore, in addition to traditional systematic reviews and living systematic reviews, we propose developing a third category: AI-driven reviews. These reviews would use AI technologies to support and potentially automate every stage, from literature search to data extraction and statistical analysis, while maintaining quality and ethical standards. Previous work has described machine-learning tools that can assist the systematic review process, and recent findings suggest that large language models may support tasks such as screening and data extraction [3,4]. In this article, we explore how AI-driven reviews could build on living systematic reviews and argue that fully AI-driven reviews have a potential role in helping researchers keep pace with the growth of scientific research.

Traditional vs. living systematic reviews

Traditionally, systematic reviews have relied on manual screening and data collection, with each step performed by two individuals. This process is often preceded by creating search strings in collaboration with information specialists to ensure capturing all relevant articles and minimizing the number of irrelevant ones. Researchers may also limit themselves to major databases like PubMed and Embase, as these are often seen as containing relevant content, sometimes leading to overlooking other databases to reduce workload.

Living systematic reviews operate differently from traditional systematic reviews in four key areas: publication format, workflow, the author team, and statistical methods [2]. The most important aspect of living systematic reviews is their publication format; they are designed to be dynamic, available online, and updated continuously, typically within short intervals [2]. The workflow includes regular updates to the literature search and the identification of new relevant studies for inclusion [2]. This leads to a continuous workload for the author team of living systematic reviews, in contrast to conventional systematic reviews, where updates occur every few years, requiring intensive and demanding work over a fixed period, sometimes taking a year or more to complete. Lastly, living systematic reviews, like traditional systematic reviews, also require assessments of bias and heterogeneity for newly included studies, along with updated statistical analyses and interpretations, to ensure they remain dynamic and relevant [2].

AI-driven reviews

AI-driven reviews could go further by aiming for full automation of all stages in the review process. By implementing advanced algorithms, it could be possible to automate literature searches across multiple databases and languages, automatically screen and select relevant studies, and perform data extraction and statistical analyses; ideally without manual intervention, although under human supervision. There has been a focus on automation over the years, and several tools have been developed to facilitate different parts of the screening and review process [3]. These tools can reduce the manual workload when updating living systematic reviews. However, the fact that the future presents opportunities to develop AI reviews means that we need tools to enhance the review process by increasing digitalization and automating multiple steps. Recent studies have also evaluated large language models across several review tasks, including search strategy development, title and abstract screening, full-text screening, and data extraction [3,4]. Overall, these tools contribute to efficiency, but there remains a need for further research to evaluate whether these separate automated steps can be combined into accurate, transparent, reproducible, and fully AI-driven review workflows. Given the rapid development and increasing capabilities of current AI systems, many of the individual tasks required for AI-driven systematic reviews may already be technically achievable when these systems are used with carefully designed prompts and tools. The main challenge may therefore be less about waiting for a completely new dedicated model and more about integrating, validating, and governing existing capabilities within a complete workflow.

Building toward full automation

With advancements in AI, there is potential for automation. Current AI systems may already support literature searching, screening, and data extraction when used within supervised review workflows and combined with carefully designed prompts and external tools [3,4]. These systems may also assist with processing and translating multilingual literature, although this application requires further validation. By using AI, we can formulate search strings and explore multiple databases and languages in a more systematic and scalable way. Natural language processing can be employed to comprehend and translate articles in different languages. Specifically, we propose striving for a higher degree of automation in systematic reviews by integrating advanced AI technologies into every step of the process:

1. Automated literature search and translation: By using AI, we can develop search strings that search across multiple databases and languages. Natural language processing allows us to process and translate articles in various languages. Tools that can assist in this process have already been described in the literature [3].

2. Automatic screening and selection of studies: Algorithms can be trained to assist in identifying relevant studies based on predefined inclusion and exclusion criteria. This can reduce the need for manually screening thousands of titles and abstracts and the subsequent full-text screening process. Promising examples have been demonstrated in studies on machine learning and large language models in systematic reviews [3,4].

3. Data extraction, bias assessment, and quality evaluation: AI tools can extract data from studies and assess their quality. Furthermore, these tools can facilitate risk-of-bias assessments and systematically evaluate the certainty of the evidence. This step of data extraction has also been demonstrated in another study. Recent studies have demonstrated promising results for data extraction, although performance varies depending on the task, prompt design, and review context [3]. Further validation is needed before these systems can be relied upon for more complex evidence appraisal tasks.

4. Statistical analyses and reporting: Current AI systems can generate and execute analytical code and interact with external tools, making it technically possible to automate data analysis and generate reports, which are updated as new data becomes available within a supervised workflow [5].

Progress has been made in the field of automating the research process. An example is the introduction of the AI Scientist [5], which has been presented as one of the first comprehensive frameworks for fully automated scientific discovery. In the paper, the researchers break down the AI Scientist’s research process into three sections: idea generation, experiment iteration, and manuscript writing. Here, they showcase the creation of a manuscript entirely driven by AI. Although it demonstrates promising results, the researchers identify limitations, which they intend to address in future versions of the AI Scientist [5].

Ethical and practical challenges

Although we are moving toward the possibility of developing fully AI-driven reviews, some ethical and practical challenges still need to be considered. It is essential to ensure that AI tools are properly trained and validated to reduce errors and biases. Additionally, ethical concerns may arise from using AI, as many AI-driven processes may lack transparency, raising the risk of algorithmic bias. Understanding these processes requires insight into the training datasets and models, which users often cannot access. Furthermore, researchers must be trained to use these AI tools to monitor the processes. Therefore, developing transparent AI models and implementing ethical guidelines for their use is important. This includes tracking decision-making processes and ensuring openness about training datasets. Moreover, interdisciplinary teams with AI and domain specialists should be used to ensure responsible implementation. One way to streamline this process would be for research groups to produce their own AI models. By using advancements such as the AI Scientist [5] or similar technologies, individual research groups or organizations such as Cochrane may be able to develop and train their own specialized AI models for evidence reviews. This approach could allow for tailored solutions that align with specific research needs. With available AI frameworks and tools, this vision is increasingly conceivable and may represent a path forward for transforming systematic reviews into fully AI-driven reviews.

Limitations

This manuscript has several limitations. First, it is a conceptual article rather than a systematic review. It does not include a formal search strategy, predefined inclusion criteria, risk-of-bias assessment, or quantitative synthesis. The discussion should therefore be interpreted as a perspective on a possible future direction for evidence synthesis rather than as a comprehensive evaluation of all available evidence. Another limitation is that the concept of AI-driven reviews is not yet an established methodological category. The term is used here to describe a proposed extension of living systematic reviews, and its practical implementation would require further methodological development, validation, and consensus. Finally, the performance of AI tools varies depending on the model, training data, prompt design, review topic, available full texts, language, and degree of human oversight. Findings from selected tools or models cannot automatically be generalized to all AI systems or all review types. In addition, because AI models are developing rapidly, evaluations of specific named models may become outdated relatively quickly. Published findings should therefore be interpreted in relation to the model versions, prompts, and tasks that were evaluated at the time of each study.

Conclusion

AI is likely to have a substantial impact on the future of research. This applies to the development of reviews, as they become automated, comprehensive, and "living". This may change the way systematic reviews are conducted. Implementing AI in the research process marks an advancement in research methodologies. Despite challenges, AI's benefits are essential, particularly for keeping evidence synthesis updated and relevant to clinical practice. The transition from traditional to living and now fully AI-driven reviews reflects this progress. Many of the individual technical capabilities required for AI-driven reviews may already be available in current AI systems, but their integration into accurate, transparent, reproducible, and appropriately supervised end-to-end workflows remains an important challenge. Fully AI-driven reviews may not merely be possible but may become necessary to manage the growth of scientific research, ensuring that reviews remain comprehensive, efficient, and directly beneficial for clinical practice.
